# Case Report and Literature Review: Intrathyroid Thymic Carcinoma

**DOI:** 10.1002/jcu.70069

**Published:** 2025-09-03

**Authors:** Jia‐Yue Sun, Ji Qi, Yi‐Lin Hou, Yun‐Fei Zhang

**Affiliations:** ^1^ Department of Ultrasound The First Affiliated Hospital of China Medical University Shenyang Liaoning China

**Keywords:** diagnosis, intrathyroid thymic carcinoma, thyroid cancer, treatment, ultrasound features

## Abstract

This case report details a case of intrathyroid thymic carcinoma (ITTC). ITTC is extremely rare with few reports. Diagnosis is difficult due to a lack of specific features and depends on pathology. A 36‐year‐old male was diagnosed. His condition and relevant tests are described. The tumor was removed, and he recovered well. This case report also discusses ITTC's characteristics, diagnosis, treatment, and prognosis, emphasizing its rarity and the need for a better understanding.

## Graphical abstract

This case report highlights the challenges in diagnosing intrathyroid thymic carcinoma (ITTC), a rare thyroid tumor, and underscores the importance of postoperative pathology for confirmation. The study emphasizes the good prognosis of ITTC with appropriate surgical management.

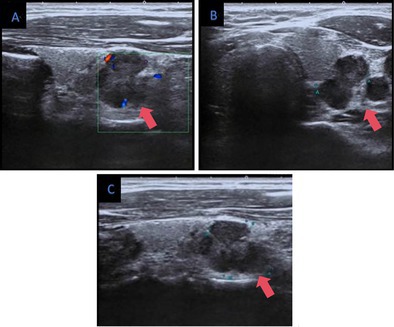

## 1 Introduction

It is believed that remnants of pharyngeal pouches capable of thymus differentiation are the cause of intrathymus epithelial thymoma (ITET)/carcinoma showing thymoid differentiation (CASTLE), a rare, low‐grade thyroid cancer that resembles lymphoepithelioma‐like and thymic squamous cell carcinomas (Dorfman et al. 1998). Miyauchi et al. (1985) first reported it as intrathyroid epithelial thymoma. Chan and Rosai (1991) classified these ectopic thymoid epithelial tumors of the neck and thyroid into four categories: ectopic hamartomatous thymoma, ectopic cervical thymoma, spindle epithelial tumor with thymus‐like differentiation, and CASTLE. The World Health Organization classification currently defines the CASTLE category as intrathyroid thymic carcinoma (ITTC) in 2004 (Chan and Rosai 1991). So far, only a few cases of ITTC have been reported.

According to studies, it is challenging to make a clinical diagnosis of ITTC, and the tumor can easily spread to other areas. Since it is challenging to differentiate ITTC from undifferentiated thyroid carcinoma, poorly differentiated thyroid carcinoma, myeloid cell carcinoma, primary squamous cell carcinoma of the thyroid gland, and metastatic thyroid carcinoma, the diagnosis of ITTC should be made using both fine needle aspiration cytology and histology (Tsutsui et al. 2013).

In this instance, the ITTC, which exhibited no overt signs, was discovered by accident during a physical examination. Our study highlights the distinctive characteristics of ITTC through in‐depth ultrasonography and pathology results. It is crucial to identify minute variations in ultrasounds since they can direct additional diagnosis. The thymic origin and biological behavior of ITTC are better understood due to our thorough pathological study, which includes immunohistochemistry markers. These results are essential for enhancing the precision of diagnosis and directing therapy. Additionally, our case highlights the importance of combining pathology, cytology, and ultrasound to obtain a definitive ITTC diagnosis. This integrative approach is essential given the complexity and rarity of the condition, emphasizing the necessity of multidisciplinary therapy for ITTC patients.

## 2 Case Report

In December 2022, a 36‐year‐old male patient was found to have a nodule in the left lobe of the thyroid gland by ultrasound examination in a local hospital, indicating a high possibility of malignancy. Ultrasound‐guided fine needle aspiration of thyroid nodules in the left lobe was performed in Huazhong University of Science and Technology Union Hospital in February 2023, and cytopathology showed Bethesda V. The patient had no pain, hoarseness, choking, pressure, dysphagia, or foreign body sensation. There was no fever, palpitation, dyspnea, hypermetabolic symptoms such as hyperphagia, emaciation, and emotional excitement, no significant changes in weight, and no family history.

### 2.1 Laboratory Tests

**FIGURE 1:**
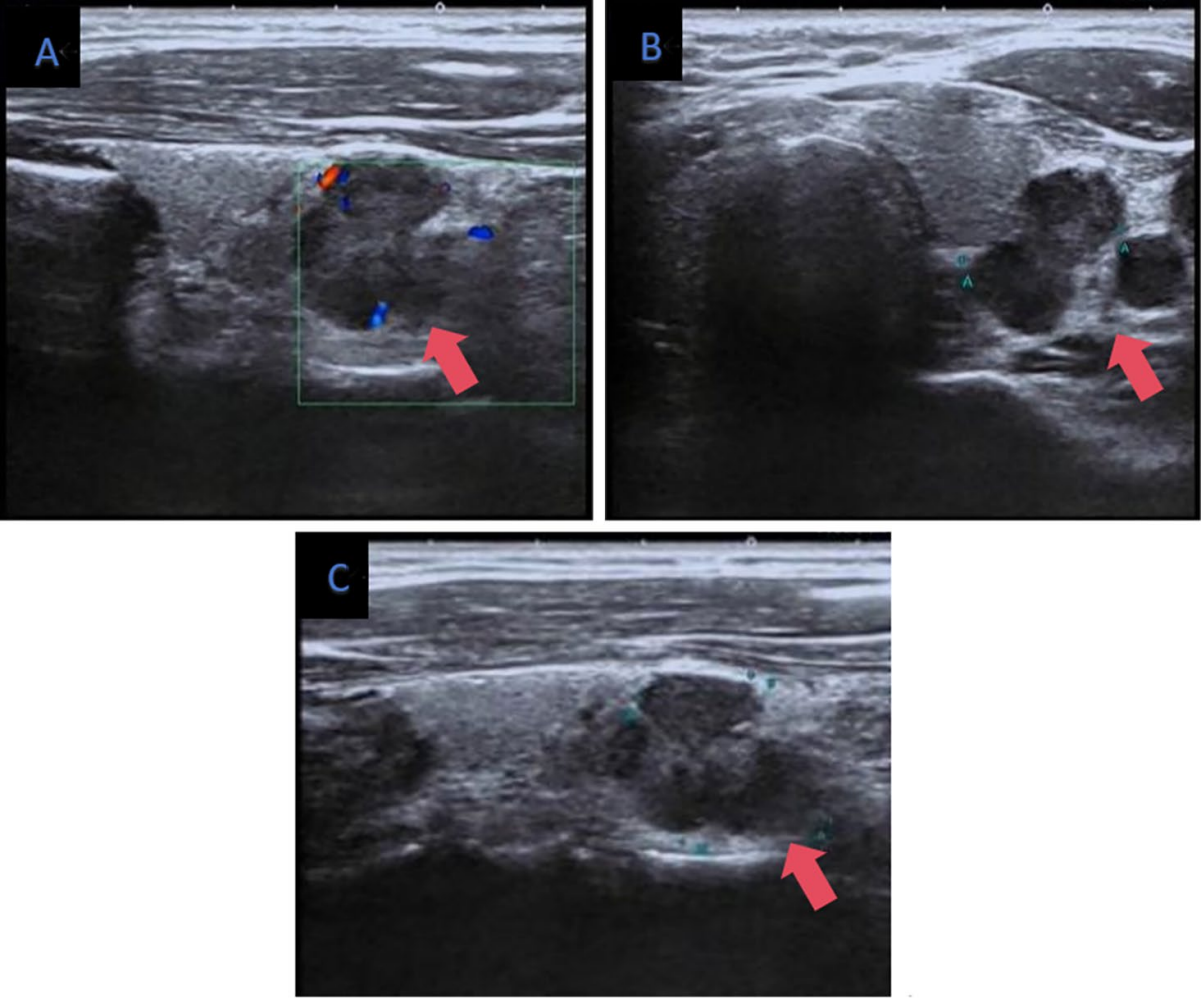
CDFI showed sparse color blood flow signals in the left thyroid lobe (A), heterogeneous hypoechoic nodules with irregular shape and well‐defined borders were seen in the left lobe (B, C) in March 2023.

**FIGURE 2:**
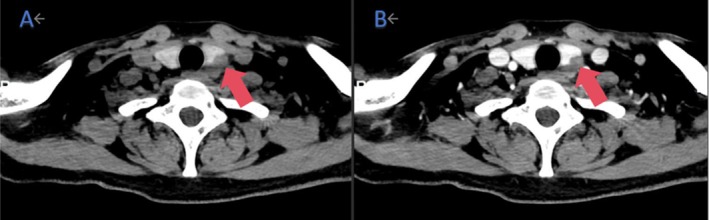
The left lobe of the thyroid gland showed a slightly low‐density shadow, and the CT value was about 48 HU on plain scan (A), with moderate enhancement in the arterial phase, the CT value about 82 HU (B) in March 2023.

Serum free thyroxine (FT4) was 14.2900 pmol/L (normal range: 9.01–19.05 pmol/L), serum free triiodothyronine (FT3) was 4.4300 pmol/L (normal range: 2.43–6.01 pmol/L), serum thyroid‐stimulating hormone (TSH) was 1.0625 mIU/L (normal range: 0.35–4.94 mIU/L), serum antithyroid microsomal antibody (TPOAb) was 2.9000 IU/mL (normal range:0.00–5.61 IU/mL), serum anti‐thyroglobulin antibody (TGAb) was 0.5400 IU/mL (normal range: 0.00–4.11 IU/mL), the serum triglyceride (TG) was 0.85 mmol/L (normal range: 0.00–1.70 mmol/L), the serum total cholesterol (TC) was 4.74 mmol/L (normal range: 0.00–5.72 mmol/L), the serum high‐density lipoprotein cholesterol (HDL‐C) was 1.38 mmol/L (normal range: 0.91–1.92 mmol/L), and the serum low‐density lipoprotein cholesterol (LDL‐C) was 3.04 mmol/L (normal range: 0.00–3.64 mmol/L), the level of 25‐hydroxyvitamin D3 (VitD3) was 21.50 ng/mL (normal range: 11.10–42.90 ng/mL). All above were within the normal range. Ultrasound examination showed a hypoechoic nodule (size: 2.14 × 1.70 × 1.56 cm) in the lower pole of the left lobe of the thyroid gland, with a clear edge and uneven internal echo. Color Doppler flow imaging (CDFI) showed sparse color blood flow signals. There were no obvious abnormal enlarged lymph nodes in both necks. Ultrasound suggested a nodule in the left lobe of the thyroid (C‐TIRADS‐4c) (Figure 1). CT examination showed a nodule shadow under the left lobe of the thyroid gland; the boundary was not clear, and the size was about 1.9 × 1.2 cm (Figure 2).

On March 20, 2023, the patient underwent “left thyroid lobe and isthmus resection and left neck VI lymph node dissection” under general anesthesia. During the operation, a hard mass with a long diameter of about 2 cm was found in the inferior outer part of the left thyroid lobe. The mass was convex and hard, and the mass was fine, with a clear boundary and intact capsule. The left recurrent laryngeal nerve was not involved. Postoperative pathological diagnosis was intrathyroid thymic carcinoma, and no carcinoma was found in lymph nodes. Intraoperative frozen pathology showed a poorly differentiated carcinoma with a nested structure and local squamous differentiation, involving parathyroid and lymphoid tissues.

### 2.2 Immunohistochemical Results

CK19 (+), synaprophysin (focal+), chromograninA (−), p27 (+), cyclin D1 (+), CK5/6 (+), P40 (+), P63 (+), galectin‐3 (+), HBNE‐I (scattered+), P53 (60% strong+), TTF‐1 (−), Tg (−), ki‐67 (20%+), calcitonin (−), CD56 (−), CD117 (+), CD5 (+), PAX8 (−).

### 2.3 Molecular Pathology Results

**FIGURE 3:**
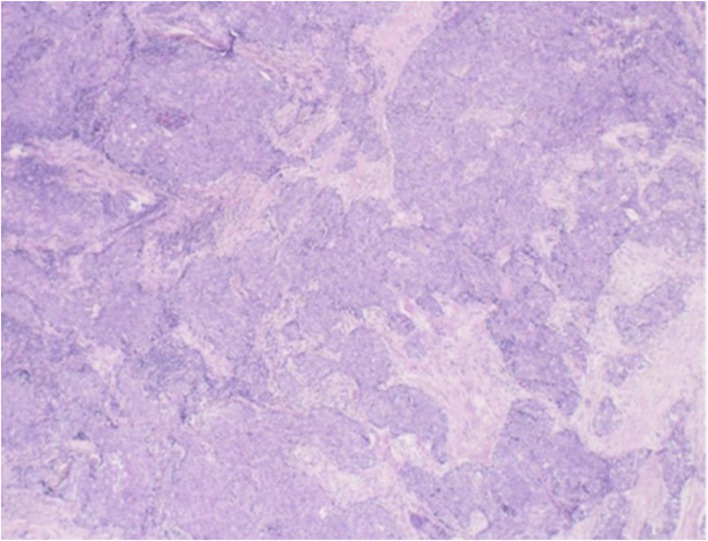
Intraoperative pathology showed a tumor in the left lobe of the thyroid gland with a nested structure and local squamous differentiation, which was diagnosed as intrathyroid thymic carcinoma.

BRAF V600e (wild type) (Figure 3), the patient recovered well after surgery, and no obvious recurrence was found by ultrasound after half a year.

## 3 Discussion

ITTC, formerly known as carcinoma with thymus‐like differentiation (CASTLE), is a rare thyroid tumor, accounting for less than 0.15% of thyroid tumors (Miyauchi et al. 1985).

In our instance, the ultrasound results showed a hypoechoic nodule in the thyroid gland's left lobe with irregular internal echoes, an irregular form, distinct boundaries, and weak blood flow signals (Figure 1). These characteristics are in line with earlier research that described ITTC as a solid mass with an irregular shape, fuzzy edges, and a hypoechoic appearance (Liang et al. 2023). In contrast to normal papillary thyroid cancer, our example showed clearer borders and sparser blood flow signs, indicating a more encapsulated development pattern. Even though it is minor, this distinction is essential for directing additional diagnostic procedures like fine‐needle aspiration. It is important to remember that ITTC's imaging characteristics on MRI, CT, and ultrasound are not unique and can mimic those of other thyroid cancers, especially thyroid carcinoma. This may help to explain why, during an ultrasound examination, ITTC may be challenging to differentiate from other thyroid tumors. Furthermore, because ITTC exhibits markedly elevated glucose metabolism on PET/CT, FDG PET/CT may be useful in the diagnosis of the condition. This can be used as an additional diagnostic tool, particularly in cases where the CT or ultrasound results are unusual (Gao et al. 2018).

The thyroid nodule's Bethesda V classification, which denotes a high suspicion of malignancy, was obtained using ultrasound‐guided fine needle aspiration cytology (FNAC). This implies that ITTC's cytological characteristics usually show up as extremely suggestive for malignancy  (Reimann et al. 2006; Mizukami et al. 1994; Hirokawa et al. 2013). In the preoperative evaluation, this cytological finding is crucial since it aids in distinguishing ITTC from other thyroid cancers. Given the challenge of differentiating ITTC from other poorly differentiated thyroid carcinomas, prior research has highlighted the significance of FNAC in conjunction with histological investigation for an accurate diagnosis (Tsutsui et al. 2013). This strategy is further supported by our instance, which emphasizes how important cytological examination is to the diagnostic method. In particular, the Bethesda V classification in our instance emphasizes how crucial it is to identify the cytological characteristics that point to a high‐grade cancer in order to direct surgical action and postoperative care.

Histological and immunophenotypic characteristics of intrathyroid thymic carcinoma (ITTC) are comparable to those of thymic carcinoma (TC), which is seen in the natural environment. With immunohistochemistry data showing CD5 (+), CD117 (+), and other markers compatible with thymic differentiation, the postoperative pathological diagnosis in this instance confirmed intrathyroid thymic cancer. These results confirm the thymic origin of ITTC and are consistent with earlier investigations that found CD5 positivity as a characteristic of the condition (Mizukami et al. 1994; Berezowski et al. 1996). Furthermore, as is typical of ITTC, the tumor displayed a layered structure with local squamous differentiation (Miyauchi et al. 1985; Kakudo et al. 2013). The tumor's involvement of the parathyroid gland and lymphoid tissue in this instance is particularly noteworthy since it highlights the aggressive character of intrathyroid thymic carcinoma (ITTC). Despite the fact that both ITTC and TC are derived from thymic epithelial differentiation, a retrospective analysis comparing the clinicopathological characteristics of nine ITTC cases and eight TC cases found that ITTC's biological behavior is less aggressive than TC's (Tahara et al. 2020). This case nevertheless emphasizes how crucial complete surgical resection is for individuals with ITTC (Liang et al. 2023; Ito et al. 2007). The diagnosis of ITTC is supported by the thorough pathological analysis, which includes the identification of particular immunohistochemical markers and offers a thorough understanding of the tumor's biological activities. This degree of specificity is essential for directing suitable treatment plans and forecasting patient results.

There is presently no proven treatment plan for intrathyroid thymic cancer (ITTC) due to its rarity. Adjuvant radiation should be explored after radical surgery in situations involving thyroid invasion and lymph node metastases. ITTC metastases to the brain, liver, and lungs have been reported; however, they are rare (Gurizzan et al. 2021; Yuan et al. 2022).

Our goal is to improve knowledge of ITTC and aid in the creation of more potent diagnostic and treatment strategies. In order to enhance patient outcomes, future research should concentrate on clarifying the molecular pathways driving ITTC and investigating targeted therapeutics.

## 4 Conclusion

In conclusion, intrathyroid thymic carcinoma is a very rare disease that is difficult to diagnose preoperatively and requires postoperative pathology for confirmation. The disease has a positive prognosis.

## Consent

Written informed consent was obtained from the individual for the publication of any potentially identifiable images or data included in this article.

## Conflicts of Interest

The authors declare no conflicts of interest.

## Data Availability

The data that supports the findings of this study are available in the Supporting Information of this article.

## References

[jcu70069-bib-0001] Berezowski, K. , M. M. Grimes , A. Gal , and M. J. Kornstein . 1996. “CD5 Immunoreactivity of Epithelial Cells in Thymic Carcinoma and CASTLE Using Paraffin‐Embedded Tissue.” American Journal of Clinical Pathology 106, no. 4: 483–486. 8853036 10.1093/ajcp/106.4.483

[jcu70069-bib-0002] Chan, J. K. , and J. Rosai . 1991. “Tumors of the Neck Showing Thymic or Related Branchial Pouch Differentiation: A Unifying Concept.” Human Pathology 22, no. 4: 349–367. 2050369 10.1016/0046-8177(91)90083-2

[jcu70069-bib-0003] Dorfman, D. M. , A. Shahsafaei , and A. Miyauchi . 1998. “Intrathyroidal Epithelial Thymoma (ITET)/carcinoma Showing Thymus‐Like Differentiation (CASTLE) Exhibits CD5 Immunoreactivity: New Evidence for Thymic Differentiation.” Histopathology 32, no. 2: 104–109. 9543665 10.1046/j.1365-2559.1998.00318.x

[jcu70069-bib-0004] Gao, R. , X. Jia , T. Ji , J. Feng , A. Yang , and G. Zhang . 2018. “Management and Prognostic Factors for Thyroid Carcinoma Showing Thymus‐Like Elements (CASTLE): A Case Series Study.” Frontiers in Oncology 8: 477. 30416983 10.3389/fonc.2018.00477 PMC6212596

[jcu70069-bib-0005] Gurizzan, C. , M. Zamparini , M. Volante , et al. 2021. “Outcome of Patients With Intrathyroidal Thymic Carcinoma: A Pooled Analysis.” Endocrine‐Related Cancer 28, no. 8: 593–604. 34105516 10.1530/ERC-21-0123

[jcu70069-bib-0006] Hirokawa, M. , A. Miyauchi , H. Minato , S. Yokoyama , S. Kuma , and M. Kojima . 2013. “Intrathyroidal Epithelial Thymoma/Carcinoma Showing Thymus‐Like Differentiation; Comparison With Thymic Lymphoepithelioma‐Like Carcinoma and a Possibility of Development From a Multipotential Stem Cell.” Acta Pathologica, Microbiologica et Immunologica Scandinavica 121, no. 6: 523–530. 10.1111/apm.12017 23176314

[jcu70069-bib-0007] Ito, Y. , A. Miyauchi , Y. Nakamura , A. Miya , K. Kobayashi , and K. Kakudo . 2007. “Clinicopathologic Significance of Intrathyroidal Epithelial Thymoma/Carcinoma Showing Thymus‐Like Differentiation: A Collaborative Study With Member Institutes of the Japanese Society of Thyroid Surgery.” American Journal of Clinical Pathology 127, no. 2: 230–236. 17210519 10.1309/VM7E52B6U9Q729DQ

[jcu70069-bib-0008] Kakudo, K. , Y. Bai , T. Ozaki , K. i. Homma , Y. Ito , and A. Miyauchi . 2013. “Intrathyroid Epithelial Thymoma (ITET) and Carcinoma Showing Thymus‐Like Differentiation (CASTLE): CD5‐Positive Neoplasms Mimicking Squamous Cell Carcinoma of the Thyroid.” Histology and Histopathology 28, no. 5: 543–556. 23233417 10.14670/HH-28.543

[jcu70069-bib-0009] Liang, J. , M. Huang , H. Huang , et al. 2023. “Intrathyroidal Thymic Carcinoma: A Retrospective Case Series Study.” Ear, Nose, & Throat Journal 102, no. 9: 584–589. 10.1177/01455613221141225 36408572

[jcu70069-bib-0010] Miyauchi, A. , K. Kuma , F. Matsuzuka , et al. 1985. “Intrathyroidal Epithelial Thymoma: An Entity Distinct From Squamous Cell Carcinoma of the Thyroid.” World Journal of Surgery 9, no. 1: 128–135. 3984364 10.1007/BF01656263

[jcu70069-bib-0011] Mizukami, Y. , A. Nonomura , T. Michigishi , et al. 1994. “Solid Cell Nests of the Thyroid. A Histologic and Immunohistochemical Study.” American Journal of Clinical Pathology 101, no. 2: 186–191. 7509563 10.1093/ajcp/101.2.186

[jcu70069-bib-0012] Reimann, J. D. R. , D. M. Dorfman , and V. Nosé . 2006. “Carcinoma Showing Thymus‐Like Differentiation of the Thyroid (CASTLE): A Comparative Study: Evidence of Thymic Differentiation and Solid Cell Nest Origin.” American Journal of Surgical Pathology 30, no. 8: 994–1001. 16861971 10.1097/00000478-200608000-00010

[jcu70069-bib-0013] Tahara, I. , N. Oishi , K. Mochizuki , et al. 2020. “Identification of Recurrent TERT Promoter Mutations in Intrathyroid Thymic Carcinomas.” Endocrine Pathology 31, no. 3: 274–282. 32594366 10.1007/s12022-020-09635-0

[jcu70069-bib-0014] Tsutsui, H. , M. Hoshi , M. Kubota , et al. 2013. “Management of Thyroid Carcinoma Showing Thymus‐Like Differentiation (CASTLE) Invading the Trachea.” Surgery Today 43, no. 11: 1261–1268. 23543082 10.1007/s00595-013-0560-2

[jcu70069-bib-0015] Yuan, Y. , C. Ke , G. Zhang , J. Zhang , and Q. Li . 2022. “Case Report and Literature Review: Thyroid Carcinoma Showing Intrathyroid Thymic Carcinoma.” Frontiers in Oncology 12: 923683. 35992819 10.3389/fonc.2022.923683 PMC9389067

